# Clinical Diagnosis has a High Negative Predictive Value in Evaluation of Malignant Skin Lesions

**DOI:** 10.1155/2021/6618990

**Published:** 2021-04-22

**Authors:** Maral Seyed Ahadi, Alireza Firooz, Hoda Rahimi, Mehrdad Jafari, Zohreh Tehranchinia

**Affiliations:** ^1^Iranian Center of Neurological Research, Neuroscience Institute, Tehran University of Medical Sciences, Tehran, Iran; ^2^Center for Research & Training in Skin Diseases & Leprosy, Tehran University of Medical Sciences, Tehran, Iran; ^3^Skin Research Center, Shahid Beheshti University of Medical Sciences, Tehran, Iran; ^4^Department of Otorhinolaryngology, Imam Khomeini Hospital Complex, Tehran University of Medical Sciences, Tehran, Iran

## Abstract

**Background:**

The increasing incidence of skin cancers in fair-skinned population and its relatively good response to treatment make its accurate diagnosis of great importance. We evaluated the accuracy of clinical diagnosis of malignant skin lesions by comparing the clinical diagnosis with histological diagnosis as the gold standard.

**Materials and Methods:**

In this retrospective study, we assessed all the pathology reports from specimens sent to a university hospital laboratory in 3 consecutive years from March 2008 to March 2010. Sensitivity, specificity, positive predictive value (PPV), negative predictive value (NPV), and positive and negative likelihood ratios were calculated for clinical diagnosis of malignant skin lesions stratified by their histological subtype.

**Results:**

A total 4,123 specimen were evaluated. The sensitivity and specificity for clinical diagnosis of malignancy were 90.48% and 82.85%, respectively, whereas the negative predictive value was shown to be 99.06%. The positive and negative likelihood ratios were 5.23 and 0.11, respectively.

**Conclusion:**

Pathological assessment of skin lesions remains the cornerstone of skin cancer diagnosis. The high NPV and the relatively low PPV indicate that clinical diagnosis is more efficient in ruling out malignancies rather than diagnosing them.

## 1. Introduction

Skin cancers, mainly constituting of malignant melanoma (MM) and nonmelanoma skin cancer (NMSC), represent the most prevalent forms of cancer in populations worldwide [1]. Based on Global Burden of Disease 2017 data, incident rate for NMSC and MM were 100.3 and 4.04 per 100,000, respectively, from which incident rates for BCC and SCC were 77.02 and 23.28 per 100,000, respectively [2]. NMSC is still a matter of great concern owing to its rising incidence over the past decade. There is a 33% (95% UI, 29%–36%) increase in NMSC cancer cases globally from 2007, 20% of which can be attributed to change in the population age structure and 13% to population growth [1]. The odds of acquiring NMSC were reported to be 1 in 7 for men and 1 in 10 for women, globally.

Incidence rates of NMSC and MM in Iran, according to GBD 2017, were 31.73 and 1.23 per 100,000, respectively [2]. Early diagnosis and optimal treatment of skin cancers greatly improve patients' outcome, thereby minimizing morbidity and mortality. Melanoma and some NMSCs, especially SCC, have the potential to metastasize and are associated with higher mortality rates, thus their early diagnosis is of vital importance. Although BCC is considered to be less dangerous, its local invasion and the consequent deformities make its early diagnosis valuable enough [3]. Clinical diagnosis is the frontier in skin cancer suspicion and diagnosis. However, its accuracy as a diagnostic test in terms of reliability and validity is not thoroughly evaluated. Sensitivity of skin cancer clinical diagnosis in different studies is variable, ranging from 56 to 97.5% [4–6]. As accurate diagnosis is the key measure for early diagnosis and treatment and consequently morbidity reduction, the aim of this study was to evaluate the accuracy of clinical diagnosis of different skin malignancies compared with pathological diagnosis as the gold standard.

## 2. Materials and Methods

In this retrospective diagnostic test study, we retrieved data from 4,236 patients undergoing incisional and excisional biopsy of skin lesions (benign or malignant) during a 3-year period at Center for Research and Training in Skin Diseases and Leprosy, a tertiary university clinic in Tehran, Iran. This study was approved by ethics committee of Tehran University of Medical Sciences.

Repeated samples from the same patient were excluded (113 samples). Variables including demographic factors (age and gender of the patients) and the anatomic region of the lesions were obtained. All of the pathology samples were evaluated and reported by the Department of Pathology at the same centers and the clinical diagnoses mentioned on pathology request forms were compared with the pathology report as the gold standard. First, the pathological diagnoses were evaluated and the specimens positive for malignancy were identified and categorized into 9 groups: squamous cell carcinoma (SCC), basal cell carcinoma (BCC), malignant melanoma (MM), lentigo maligna (LM), malignant adnexal tumors (MAT), Bowen's disease, Kaposi's sarcoma, Paget's disease, and mycosis fungoides (MF). Afterwards, the proposed clinical differential diagnoses were evaluated; if the first diagnosis was consistent with the pathological diagnosis, it was recorded as to be correct, if not, the first diagnosis was evaluated to be benign or malignant and if the correct diagnosis was considered in the differential diagnosis or not. Other specimens negative for malignancy by pathological diagnosis were considered negative based on the gold standard.

In order to calculate the sensitivity, specificity, positive predictive value (PPV), negative predictive value (NPV), and positive and negative likelihood ratios (LR), we cross-tabulated the clinical diagnoses against the pathological diagnoses (the gold standard) and calculated the formers based on standard formulae. We used SPSS Statistics 20.0 for Windows (SPSS Inc. Chicago, IL, USA) software package for analysis of the data.

## 3. Results

Out of 4,236 samples, 113 were excluded from the study based on exclusion criteria. Of the remaining 4,123 samples, 2,253 (54.7%) belonged to female patients. The mean age of the patients was 47.6 ± 18.6 years (44.5 ± 18.9 for women and 50.8 ± 17.9 for men). From the 4,123 samples, 938 were clinically suspicious for malignancy, from which 285 were confirmed by histopathology (true positive), while 653 were not recognized as malignant in histopathology (false positive). From the 3,185 clinically benign diagnosed lesions, 30 were pathologically malignant (false negative) and 3,155 were reported as both clinically and pathologically benign (true negative).  Table 1 illustrates the accuracy of clinical diagnosis based on the first proposed differential diagnosis.

The most common malignant tumor confirmed by pathology was BCC (202 samples), accounting for 64.1% of the malignant tumors, followed by mycosis fungoides (35 samples), malignant adnexal tumors (29 samples), SCC (23 samples), Bowen's disease (12 samples), malignant melanoma (5 samples), lentigo maligna (5 samples), Paget's disease (3 samples), and KS (1 sample).

Malignant lesions were anatomically distributed as follows: 2,468 head and neck cases (59.8%), 708 trunk cases (17.2%), 298 upper limbs cases (7.2%), and 394 lower limbs cases (9.6%).

Sensitivity and specificity of clinical diagnosis for any skin malignancy were 90.48 (95% CI: 87.24–93.72) and 82.85 (95% CI: 81.66–84.04), respectively. Positive and negative predictive values for all types of skin cancers were 30.38 (95% CI: 27.44–33.32) and 99.06 (95% CI: 98.73–99.39), respectively. In other words, 30.38% of patients with a clinical diagnosis of skin cancer actually had the disease and 99.06% of patients with clinical diagnosis of benign skin lesions were truly free of malignancy.  Table 2 depicts sensitivity, specificity, PPV, and NPV of clinical diagnosis for malignancy based on histopathological subtypes. Tables 3 and 4 illustrate sensitivity and PPV of malignant lesions based on anatomical distribution, respectively. Furthermore, positive and negative likelihood ratios of clinical diagnosis for skin malignancy were calculated as 5.28 and 0.11, respectively.

## 4. Discussion

In the present study, 4,123 specimens were evaluated for malignancy from which 315 malignant cases were confirmed by pathology. The highest and lowest sensitivity for clinical diagnosis of malignancy was for BCC and Bowen, respectively (91.58% vs. 25%), whereas malignant adnexal tumors and BCC had the highest and lowest specificity, respectively (99.61 vs. 93.01).

Clinical diagnosis is the cornerstone of suspicion for malignancy where other diagnostic utilities would then be applied to evaluate further. It is of clinical importance to have an estimation of the frontier of malignancy diagnosis. Sensitivity and specificity are two measures of particular interest in the literature. Heal et al. in a study in 2008 reported a sensitivity of 56% for combination of SCC and BCC as NMSC [5]. They also estimated PPV of SCC, BCC, and MM to be 49.4%, 72.7%, and 33.3%, respectively. However, as their sample population was recruited from cancer clinics with higher prevalence of malignancy (74.82% vs. 7.64 in the present study), comparison of the studies is not impeccable.

In another study by Cooper and Wojnarowska, sensitivity for SCC and BCC was estimated to be 59% and 66.6%, respectively [7]. They also reported a specificity of 75.3% and 85.6% for SCC and BCC, respectively. We reported a higher specificity for clinical diagnosis of SCC and BCC compared to the aforementioned studies. However, we need an index to predict if a clinical diagnosis indicates malignancy; what the odds are that the lesion is truly malignant. In order to estimate that we require pretest probability, which is proportionate to prevalence and in combination with sensitivity and specificity can be used to measure positive and negative likelihood ratios. Afterwards, posttest probability can be estimated with the application of a nomogram.

Likelihood ratios are alternative statistics for summarizing diagnostic accuracy, which have several particularly powerful properties that make them more useful clinically than other statistics. It is advantageous in comparison to indices such as sensitivity and specificity that it combines the data from sensitivity and specificity into one single index and it is not affected by prevalence which is another advantage facilitating its application in practice. Likelihood values of a diagnostic test greater than 10, for not a rare disease, predict a high probability of disease, whereas values below 0.1 can be a good predictor of being ruling out disease when the likelihood ratio is negative [8]. In the present study, the estimated positive likelihood ratio for clinical diagnosis of malignancy was estimated to be 5.28 and when taking into account the prevalence of malignancy in our sample (7.64%), it would result in a posttest probability of disease of less than 30%. Accordingly, with a clinical diagnosis of malignancy, the probability of the patient to actually have malignancy would be less than 30%, which is in line with the estimated PPV. Furthermore, we estimated a negative likelihood ratio of 0.1% indicating that if a patient is clinically diagnosed not to have skin cancer, the probability of the diagnosis to be wrong is less than 0.1%. This is to emphasize that clinical diagnosis is more powerful in ruling out skin cancers than diagnosing them. Therefore, it seems that it is an efficient tool for malignancy screening.

In a study by Nault et al. [9], numbers needed to biopsy (NNB) for all skin cancers and melanoma were reported to be 3.4 and 21.4, respectively. In another recent meta-analysis by Nelson et al., NNB for melanoma was estimated from studies published between 2000 and 2018 which was 15.6 worldwide [10]. In the present study, NNB was 3.29 and 27.24 for diagnosis of all skin malignancies and melanoma, respectively. As our study sample is not from a tertiary skin cancer clinic, resultant NBB is comparable with the study performed by Nault et al. which is also not from cancer clinics.

Another outcome of this study is that by comparing the first differential diagnosis with pathological diagnosis, we can estimate the necessity of sample acquisition. It can be concluded that even though the physician has not diagnosed the malignant lesion correctly, nevertheless, they have diagnosed the lesion as malignant at the top of the differential diagnosis in 77.14% of cases and have excised the lesion properly. However, in 4.76% of the cases, the malignancy had not been diagnosed at all. In a study by Matteucci et al. [11], sensitivity and specificity of malignancy diagnosis, irrespective of the exact histological subtype, were 91% and 84%, which is comparable with our results (90.48% and 82.85%, respectively).

This study is limited by the fact that the studied sample is not the exact representative of the skin specimens negative for malignancy and only includes samples that needed biopsy in order to have an accurate diagnosis and results in a verification bias. Though it cannot be corrected due to ethical issues, the direction of variations could be accounted for properly. Consequently, sensitivity is overestimated and specificity, NPV, and negative likelihood ratio indices are underestimated, which further emphasizes that clinical diagnosis is efficient in ruling out malignancies. Another limitation is that due to retrospective nature of the study, it is not possible to account for the physicians' uncertainty in diagnosis of skin cancers; some of the samples maybe biopsied due to national protocols or patients' preference increasing false negative samples. Finally, in this study, we evaluated clinical diagnosis of skin cancers by naked eye examination. However, dermoscopy may increase sensitivity of clinical diagnosis for NMSCs and melanoma and is widely incorporated into daily practice [12–14].

## 5. Conclusion

Biopsy of skin lesions remains the cornerstone of skin cancer diagnosis. The high NPV and the relatively low PPV indicate that clinical diagnosis is more efficient in ruling out malignancies rather than diagnosing them.

## Figures and Tables

**Table 1:** Accuracy of clinical diagnosis based on first differential diagnosis.

Clinical diagnosis		*N* (percent)
Accurate diagnosis		223 (70.79%)
Accurate diagnosis in DDX, first diagnosis benign		49 (15.56%)
Diagnosis of malignancy, accurate diagnosis in DDX		9 (2.86%)
Accurate diagnosis not in the DDX	First diagnosis benign	15 (4.76%)
	First diagnosis malignant	11 (3.49%)
	No DDX mentioned	8 (2.54%)
Total		315 (100%)

**Table 2:** Sensitivity, specificity, positive predictive value, and negative predictive value of clinical diagnosis based on malignancy subtype.

	Sensitivity	Specificity	PPV	NPV
SCC *N* = 23	36 (17.19–54.81)	98.29 (97.90–98.68)	11.39 (4.39–18.39)	99.65 (99.47–99.83)
BCC *N* = 202	91.58 (87.76–95.40)	93.01 (92.23–93.79)	50.55 (45.43–55.67)	99.55 (99.34–99.76)
Bowen *N* = 12	25 (0.5–49.5)	99.57 (99.37–99.77)	15 (0–30.64)	99.78 (99.64–99.92)
Kaposi's sarcoma *N* = 1	100 (2.50–100)	99.20 (98.93–99.47)	2.94 (0–8.61)	100 (99.91–100)
Paget's disease *N* = 3	100 (29.24–100)	100 (99.91–100)	100 (29.24–100)	100 (99.91–100)
Malignant melanoma *N* = 5	80 (44.94–100)	97.45 (96.97–97.93)	3.67 (0.15–7.19)	99.97 (99.92–100)
Lentigo maligna *N* = 5	40 (0–82.94)	99.34 (99.10–99.58)	6.90 (0–16.12)	99.93 (99.85–100)
Mycosis fungoides *N* = 35	91.43 (82.16–100)	95.01 (35.94–95.67)	13.56 (9.20–17.92)	99.92 (99.84–100)
Malignant adnexal tumors N = 29	86.21 (73.67–98.75)	99.61 (99.42–99.80)	60.97 (46.04–75.90)	99.90 (99.81–99.99)

**Table 3:** Sensitivity of clinical diagnosis of malignant lesions and 95% confidence intervals based on anatomical site.

Clinical diagnosis	Head and neck	Trunk	Upper extremity	Lower extremity
SCC	30 (9.92–50.08) *N* = 20	—	100 (2.5–100) *N* = 1	100 (15/81–100) *N* = 2
BCC	92.35 (88.50–96.20) *N* = 183	85.71 (59.79–100) *N* = 7	50 (1.26–98.74) *N* = 2	0 (1.25–84.18) = 1
Bowen	16.66 (0–46.47) *N* = 6	25 (0–67.43) *N* = 4	50 (1.26–98.74) *N* = 2	—
Malignant melanoma	0 (1.25–84.18) *N* = 1	100 (2.5–100) *N* = 1	100 (15.81–100) *N* = 2	100 (2.5–100) *N* = 1
Lentigo maligna	40 (0–82.94) *N* = 5	—	—	—
Kaposi's sarcoma	—	—	—	100 (2.5–100) *N* = 1
Mycosis fungoides	66.66 (13.32–100) *N* = 3	90 (71.41–100) *N* = 10	100 (29.24–100) *N* = 3	83.33 (53.51–100) *N* = 6
Malignant adnexal tumors	85.18 (71.78–98.58) *N* = 27	—-	—	—
Paget's disease	—	100 (29.24–100) *N* = 3	—	—

**Table 4:** Positive predictive value of clinical diagnosis of malignant lesions and 95% confidence intervals based on anatomical site.

Clinical diagnosis	Head and neck	Trunk	Upper extremity	Lower extremity
SCC	11.32 (2.79–19.85) *N* = 20	—	16.67 (0.42–64.12) *N* = 1	18.18 (2.28–51.78) *N* = 2
BCC	51.06 (45.68–56.44) *N* = 183	37.50 (15.20–64.27) *N* = 7	50 (9.45–90.55) *N* = 2	0 (1.25–84.18) *N* = 1
Bowen	14.28 (0–40.19) *N* = 6	16.66 (0.42–64.12) *N* = 4	50 (9.45–90.55) *N* = 2	—
Malignant melanoma	0 (0–84.18) *N* = 1	4.35 (0.11–21.95) *N* = 1	11.76 (0–27.07) *N* = 2	5.88 (0.15–28.69) *N* = 1
Lentigo maligna	9.52 (0–22.07) *N* = 5	—	—	—
Kaposi's sarcoma	—	—	—	8.33 (0–23.96) *N* = 1
Mycosis fungoides	10 (0–23.14) *N* = 3	9.78 (4.57–17.76) *N* = 10	7.89 (1.66–21.38) *N* = 3	9.43 (1.56–17.30) *N* = 6
Malignant adnexal tumors	63.88 (48.19–79.57) *N* = 27	—	—	—
Paget's disease	—	100 (29.24–100) *N* = 3	—	—

## Data Availability

The data used to support the findings of this study are included within the article.
